# How general pediatricians learn procedures: implications for training and practice

**DOI:** 10.1080/10872981.2021.1985935

**Published:** 2021-10-13

**Authors:** Maya S. Iyer, David P. Way, Daniel J. Schumacher, Charmaine B. Lo, Laurel K. Leslie

**Affiliations:** a Director of Emergency Medicine Faculty Development, Nationwide Children’s Hospital, Columbus, Ohio; b Department of Pediatrics, The Ohio State University College of Medicine, Columbus, Ohio; c Department of Emergency Medicine, The Ohio State University College of Medicine, Columbus, Ohio; d Department of Pediatrics, Cincinnati Children’s Hospital, Cincinnati Ohio, USA; e Abigail Wexner Research Institute at Nationwide Children’s Hospital, Columbus, Ohio; f The American Board of Pediatrics, Chapel Hill, North Carolina, USA; g Department of Pediatrics, Tufts School of Medicine, Boston, MA, US

**Keywords:** Accreditation council for graduate medical education, pediatrics, residency, procedures, Education, mastery learning

## Abstract

The Accreditation Council for Graduate Medical Education (ACGME) requires General Pediatricians (GPeds) to learn thirteen procedures during training. However, GPeds infrequently perform these procedures in practice. We sought to determine:1) how GPeds learned procedures, 2) if GPeds self-reported achieving competence in the required ACGME procedures during training, and 3) if GPeds maintained these skills into practice. We conducted this mixed methods study from 2019–2020. 51 GPeds from central Ohio and the American Board of Pediatrics General Examination Committee were recruited via email or snowball sampling and participated in semi-structured recorded phone interviews probing procedural performance during training and current practice. Participants represented varied geographic regions and clinical settings. We employed Sawyer’s ‘Learn, See, Practice, Prove, Do, Maintain’ mastery learning pedagogical framework as a lens for thematic analysis. Participants did not demonstrate competence in all ACGME required procedures during training, nor sustain procedural skills in practice. Most participants learned procedures through a ‘see one, do one’ apprenticeship model. GPeds reported never being formally assessed on procedural competence during residency. All GPeds referred out at least one procedure. GPeds also believed that skill maintenance was unwarranted for procedures irrelevant to their current practice. GPeds did not sufficiently demonstrate competence in all ACGME required procedures during training, partially suggesting why they infrequently perform some procedures. Alternatively, these required procedures may not be relevant to their practice. Pediatric residency procedures education might consider using mastery learning for practice-specific procedures and surface-level methods (learning without mastery) for other skills.

## Introduction

Therapeutic procedures, once considered the surgeon’s exclusive domain, are now practiced in almost all medical specialties[1]. Even though general pediatrics is not known to be a ‘procedures-heavy’ discipline, general pediatricians (GPeds) are expected to provide a medical home for their patients, which includes performing common procedures safely and effectively [2–6]. For nearly 30 years, common pediatric procedures have been incorporated into residency program curricula in the USA[7]. In 2006, the Accreditation Council for Graduate Medical Education (ACGME) introduced procedure requirements – compulsory skills that residents must competently perform prior to graduation. Competence is ‘individual characteristics (knowledge, abilities and attitudes) that allow a person to practice an activity in an autonomous fashion, to continuously improve practice and to adapt to a rapidly [changing] environment.’[8] Demonstrating competence requires a trainee to ‘show how’ they are sufficiently skilled to perform a procedure[9]. While the ACGME requires that graduates demonstrate competence in required procedures, they leave it up to the discretion of residency programs to determine core methods of competence assessment, often with input of program specific clinical competency committees[10]. Table 1 displays the most recent list of ACGME required procedures for pediatric residents[11].

**Table 1.** The current procedures recommended by the ACGME for pediatric residency programs

Emergent Procedures	Urgent Procedures	Office-Based Procedures
Bag-Valve Mask Ventilation Neonatal Endotracheal Intubation Umbilical Catheter Placement	Lumbar Puncture Simple Laceration Repair Incision & Drainage of Abscess Reduction of a Dislocation Temporary Splinting of a Fracture	Giving Immunizations Bladder Catheterization Peripheral Intravenous Catheter Placement Venipuncture Removal of a Foreign Body

Learning procedures under mastery learning models is effective in both procedure heavy disciplines, such as surgery, as well as primary care specialties such as internal medicine, pediatrics, or obstetrics and gynecology [12–15]. Mastery learning models that incorporate deliberate practice, the process of performing the skill under direct observation of an instructor with immediate formative feedback, are favored to traditional frameworks such as the common apprenticeship ‘see one, do one’ technique that only requires learners to demonstrate rudimentary skill [16,17]. Deliberate practice builds learners’ skills until they have achieved a level of performance that is comparable to that of a master. Training to mastery levels of performance is effective because it reliably predicts future skill performance resulting in optimal health care outcomes, whereas minimum competency learning, a potential outcome of ‘see one, do one’ education, leads to rapid skill performance deterioration (decay) [18–20].

**Figure 1.**
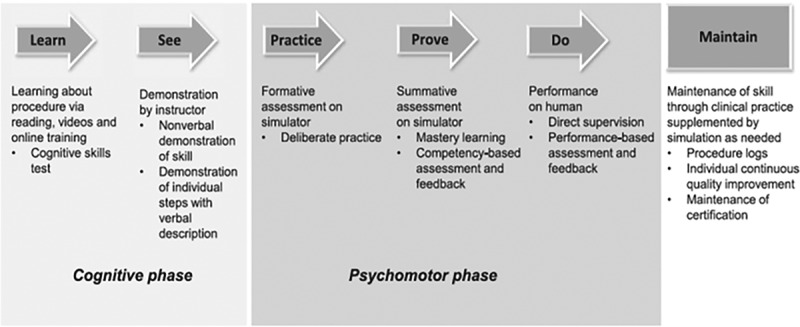
Sawyer’s learn, see, practice, prove, do, maintain pedagogical framework for procedural skill training in medicine

Sawyer’s ‘*Learn, See, Practice, Prove, Do, Maintain’* pedagogical framework is one such mastery learning model for learning procedures (Figure 1)[21]. Prior to ‘seeing’ a procedure, this model requires trainees to first learn the foundational underpinnings of what a procedure entails (the steps, indications, contraindications, complications, pain management, post-procedure care, and interpretation of applicable results) through readings, didactics, or web-based modules [11,21]. Then, trainees see demonstrations of the procedure and engage in efforts to model the collective tasks that comprise the procedure through deliberate practice and feedback[17]. At the point during the deliberate practice stage where individual learners believe they are ready, the learner advances to the ‘prove’ phase of Sawyer’s model. Once they have demonstrated competence at the ‘prove’ phase, they are considered qualified to perform the procedure under supervision in the clinical setting. As learners progress through these stages, direct supervision gradually decreases until learners reach unsupervised performance. The final phase of Sawyer’s framework involves the maintenance of procedural skills over time.

**Reference**: Sawyer T, White M, Zaveri P, Chang T, Ades A, French H Learn, see, practice, prove, do, maintain: an evidence-based pedagogical framework for procedural skill training in medicine. Acad Med. 2015 Aug;90(8):1025–33.

From prior studies, we know that GPeds do not perform most of the currently required ACGME procedures in their practice for a variety of reasons including lack of supplies or personnel, time constraints, decreased self-confidence, decreased clinical opportunities, or reimbursement barriers [22–24]. GPeds also suggested that they may not have learned all procedures to a level of competence during training[24]. We wondered whether current GPeds’ disinclination to perform procedures was associated with the later, particularly how they learned procedures and if they received procedural competency assessments during residency. Using Sawyer’s model as a conceptual framework for evaluating procedural learning, the objectives of this study were to determine: 1) how GPeds learned procedures, 2) if GPeds self-reported achieving competence in the required ACGME procedures during training, and 3) if GPeds maintained these skills into practice.

## Materials and methods

### Setting and participants

The target population was GPeds who completed residency and currently practice in the USA. We drew our sample from two sources: a database containing the names and contact information for GPeds practicing throughout central Ohio [22,23] and the roster for the American Board of Pediatrics (ABP) General Pediatrics Examination Committee. The central Ohio GPeds database represented a range of practicing GPeds and allowed us to effectively sample GPeds who practiced in urban, suburban, and rural settings. Oversampling GPeds from rural settings was particularly critical for us since these practitioners generally self-report performing more procedures than those in suburban and urban settings [22]. We used snowball sampling from the central Ohio GPeds database to increase rural GPeds representation [24]. The inclusion of members of the ABP General Examination Committee provided more breadth of practicing GPeds throughout the USA, as the ABP chooses pediatricians from academic and community settings and diverse training backgrounds. We ensured that sampled GPeds practiced across urban, suburban, and rural geographic settings as defined by proximity to a Level 1 or 2 pediatric trauma center (PTC) (Urban: <10 miles; Suburban: 10–30 miles; and Rural >30 miles from a PTC). We sent central Ohio GPeds a postcard, followed by a telephone call or email to schedule an interview. Members of the General Pediatrics Examination Committee were recruited through an email from the ABP.

### Design, measures, and data collection

We employed a convergent parallel mixed method design [25,26] with an original semi-structured interview combined with a close-ended question instrument (Appendix A). Specifically, we asked GPeds’ about their residency training and how they learned and performed the 13 required ACGME procedures, their level of skill to perform these procedures at graduation and their current skill level, their opinions about maintenance of procedural skill over the course of their careers, how they manage procedures in their current practice, and their practice type and distance from a Level 1 or 2 PTC. We piloted these instruments among general pediatricians who did not participate in the study to ensure clarity and understanding. We conducted interviews until we achieved thematic and geographic sufficiency [26–28]. We provided a $50 MasterCard© to each interviewee for their participation.

**IRB Statement**: The study was deemed exempt by the institutional review board.

## Data analysis

We used Sawyer’s model as a framework for qualitative data interpretation and Braun and Clarke’s six steps of thematic analysis to guide our analytic approach[29]. Our qualitative analysis involved intensive group discussion among three study team members (MSI, DPW, CBL) for familiarizing us with the data, generating initial codes across all data, identifying and reviewing themes, and creating a thematic map prior to developing a codebook. These study team members met to code five randomly selected transcripts, following Braun and Clarke’s six-step process. After development of the codebook, we divided the remaining transcripts such that two of these three study members coded each individual transcript. When the two coders disagreed, a discussion was held with all three members to negotiate agreement. All study authors reviewed the final themes to ensure appropriate representation.

For the closed ended questions, we converted responses to numbers and analyzed them with descriptive statistics (means, standard deviations, frequencies and percentages). We used paired t-tests to compare GPeds self-reported level of skill performance at residency graduation to their current performance. This data was analyzed using SPSS (IBM Corp. Released 2019. IBM SPSS Statistics for Windows, Version 26.0. Armonk, NY: IBM Corp).

## Results

From June 2019 to January 2020, 51 GPed (40 from central Ohio and 11 from the ABP) participants completed the survey and interview. Interviews averaged 33 minutes in length (range: 23–96 minutes).

### Participant and practice characteristics

Most of the participants were female (64.7%; n = 33) and more than half (56.8%; n = 29) had been practicing for greater than 11 years. Most (80.4%; n = 41) worked full time and practiced an average of 30.9 miles (STD = 28.4) from a tertiary medical care center. Participants represented 26 different residency programs throughout the USA. There were no differences in themes extracted between Central OH GPeds’ and ABP General Examination GPeds’ responses. Three participants reported completing more procedures because of their training location (one in an urban setting and two in a rural setting). Otherwise, there were no associations between procedures learned and residency program setting. Practice characteristics of participants varied widely and there was equal representation of urban, suburban, and rural GPeds. Participants represented a diversity of practice types, with most (n = 41 of 51; 80.4%) spending an average of 80.5% in an office/private practice setting (Table 2). Every GPed said that they currently refer at least one procedure to emergency departments, hospitals, or outpatient subspecialists, regardless of their own practice type (academic medical center, hospital, private practice).

**Table 2.** Demographic and practice profile with frequencies (counts) and percentages of the 51 general pediatricians who participated in interviews about how they learned procedures. †Type of current practice is reported as the number of general pediatricians who work in each type of practice and the average percentage of time they allocate to that practice type

Demographic characteristic		N (%)
Female		33 (64.7)
Number of Years in Practice as GPeds	< 5	12 (23.5)
	5–10	10 (19.6)
	11–20	18 (35.3)
	21–30	8 (15.7)
	>30	3 (5.9)
Pursued Subspecialty Training		6 (11.8)
Work Full Time		41 (80.4)
Other providers (MD, DO, NP, PA) in GPed’s practice		46 (90.2)
Location of Residency Training*	Northeast	6 (11.7)
	Southern	4 (7.8)
	Central	36 (70.5)
	Western	5 (9.8)
Location of Current Practice*	Northeast	4 (7.8)
	Southern	3 (5.9)
	Central	42 (82.4)
	Western	2 (3.9)
Setting of Current Practice	Urban	14 (27.4)
	Suburban	20 (39.2)
	Rural	17 (33.3)
		N (Average %of Time)
Type of Current Practice†	Office/private practice	41 (80.5)
	Hospital Medical Center	10 (35.5)
	Emergency Department	7 (37.9)
	Newborn Nursery	14 (27.9)
	Labor and Delivery	4 (19.5)
	Urgent Care	3 (13.3)
	Federally Qualified Health Ctr	1 (100)
	Other Setting	13 (43.7)
	- Research	1 (38.5)
	- Informatics	1 (90.0)
	- Administration/Education	11

*Association of American Medical Colleges Regional Categories, December 2020

*Abbreviations: MD = Doctor of Medicine; DO = Doctor of Osteopathic Medicine; NP = Nurse Practitioner; PA Physician Assistant*

*Abbreviations: MD = Doctor of Medicine; DO = Doctor of Osteopathic Medicine; NP = Nurse Practitioner; PA Physician Assistant*

### Reported procedural learning experiences and preparedness

Nearly 10% of participants described having little experience in performing the 13 required ACGME procedures, aside from umbilical catheter placements and lumbar punctures. More than 50% of the participants said they never gave immunizations, placed a temporary splint, or inserted a peripheral IV during training. None of the participants reduced a dislocation other than a nursemaid’s elbow. Table 3 shows the number of GPeds who had limited experience for each required procedure with quotations that highlight their lack of experience in learning these skills.

**Table 3.** Number (N) and percentage (%) of GPeds with little to no experience performing the procedure during residency training along with representative quotes that provide explanation for their lack of experience

Procedure	Number N () Participants Commenting Little to No Experience	Representative Quote
Bag Mask Ventilation	7 (13.7)	*‘Bag valve mask ventilation was mostly through simulation … the only time that I was exposed to doing bag valve mask ventilation was in the NICU during resuscitation and not super frequently.’* *‘I can’t say that I remember a time when I actually bagged a[n] older kid.’*
Neonatal Endotracheal Intubation	5 (9.8)	*‘I can’t say that I’ve ever successfully done this on a real person. I put like a couple LMAs, but I never actually intubated a live person.’* *‘I did intubate some babies in the NICU [but] I never had more than one attempt.’*
Umbilical Catheter Placement	3 (5.9)	*‘I was kind of a witness to one. I don’t think overall this is something that we do repeatedly.’* *‘I learned this as well in a skill lab during my residency training and probably had like three attempts throughout my residency career to do the umbilical catheter placement.’*
Lumbar Puncture	3 (5.9)	*‘I don’t know that I have a lot of vivid memories of [learning] these.’* *‘I probably did only two lumbar punctures.’*
Simple Laceration Repair	12 (23.5)	*“I honestly don’t feel like I ever really learned that, which I should have but I really never did …* *‘In residency, the only time we got to do that was in the emergency room and even then, we didn’t get to do it very often because they had so many suture techs working.’*
I & D of abscess	10 (19.6)	*‘Honestly, I don’t think I ever did that because the suture techs always did that.’* *‘Not a lot, not as many as I wanted to just because it’s always like hit or miss with your emergency department shifts, but I felt like that’s something I could do in my office. But I don’t really feel comfortable with it.’*
Reduction of a Simple Dislocation	51 (100)	*‘Reduction … that is probably the one I’m least comfortable with because I really didn’t do a ton of that at all. I’ve never even done a nursemaid’s yet.’* *‘I did like two or three nursemaid’s. I never fixed a dislocated joint or anything like that. I definitely wasn’t trained on any of that.’*
Temporary Splinting of a Fracture	18 (35.3)	*‘I can tell you I never actually did a temporary splint of a fracture.’* *‘I think I was supposed to learn this in the ED, but don’t think I did, and I have no idea how to do it. I didn’t learn anything or definitely did not get enough formal practice.’*
Giving Immunizations	24 (47.1)	*‘I don’t think I was ever trained on giving immunizations. I mean no one said “Here’s the thing. Go ahead and give it.” I mean I knew what to do, but I don’t think I was every trained.’* *‘I never gave a shot as a resident, never.’*
Bladder Catheterization	12 (23.5)	*‘I did not and still do not have a lot of experience cathing [sp].’* *‘I would assess the need and order it, but I never placed a bladder catheterization.’*
Peripheral Intravenous Catheter Placement	23 (45.1)	*‘I remember attempting some IVs as a resident. I don’t recall if I was ever successful at doing one.’* *‘IV placements? I wish we would have gotten more of because we didn’t get a whole lot of practice with that.’*
Venipuncture	17 (33.3)	*‘I actually probably did more of those as a med student rather than a resident. As a resident, we really didn’t do venipunctures.’* *‘I did arterial punctures. This is just venipuncture? I think I did maybe a couple.’*
Removal of a Foreign Body	13 (25.5)	*“I don’t recall ever doing unless it was something like in the ear canal which I thought I could get it out by forceps or something like that. Nasal canal that I could get to. So, a couple times. And definitely not frequently*. *‘Yeah. I honestly don’t remember ever having the opportunity to remove a foreign body from anything.’*

*Abbreviations: LMA = Laryngeal Mask Airways; NICU = Neonatal Intensive Care Unit; NRP = Neonatal Resuscitation Program; PALS = Pediatric Advanced Life Support; PICU = Pediatric Intensive Care.*

GPeds also reported feeling less prepared in practice than they did at graduation across five specific procedures: neonatal endotracheal intubations, umbilical catheter placements, lumbar punctures, simple laceration repairs, and peripheral IV placements (Table 4). For these five procedures, not only were their self-reported ratings of preparedness significantly lower now than their personal ratings at graduation, but the effect sizes related to these differences were moderate to large[30].

**Table 4.** Ratings of preparedness to perform procedures (now and at residency graduation) from 39 general pediatricians

Procedure (n)	Setting	Mean	Std. Dev.	t	df	p	es*
Bag Mask Ventilation (39)	Residency	2.90	0.31	0.63	38	0.53	0.124
	Practice	2.85	0.49				
Neonatal Endotracheal Intubation (39)	Residency	2.33	0.62	2.84	38	0.007	0.537
	Practice	1.95	0.79				
Umbilical Catheter Placement (38)	Residency	2.34	0.75	5.72	37	<0.001	0.983
	Practice	1.61	0.76				
Lumbar Puncture (39)	Residency	2.87	0.34	5.18	38	<0.001	0.927
	Practice	2.31	0.73				
Simple Laceration Repair (39)	Residency	2.62	0.59	3.41	38	0.002	0.593
	Practice	2.21	0.77				
Incision & Drainage of Abscess (38)	Residency	2.45	0.69	−0.77	37	0.45	−0.118
	Practice	2.53	0.65				
Reduction of Dislocation (34)	Residency	1.82	0.80	−0.50	33	0.62	−0.103
	Practice	1.91	0.90				
Temporary Splinting (39)	Residency	2.03	0.74	−0.50	38	0.62	−0.100
	Practice	2.10	0.79				
Giving Immunizations (38)	Residency	2.24	0.75	−1.36	37	0.18	−0.221
	Practice	2.39	0.68				
Bladder Catheterization (39)	Residency	2.51	0.64	1.56	38	0.13	0.259
	Practice	2.33	0.74				
Peripheral Intravenous Catheter Placement (39)	Residency	2.10	0.72	2.82	38	0.008	0.468
	Practice	1.77	0.71				
Venipuncture (39)	Residency	2.21	0.62	1.30	38	0.20	0.186
	Practice	2.08	0.74				
Remove Foreign Body (39)	Residency	2.44	0.60	−2.16	38	0.04	−0.408
	Practice	2.67	0.53				

*Preparedness scale: 1 = unprepared, 2 = somewhat prepared, 3 = well prepared. Comparisons over time were performed with dependent (paired) t-tests.*

*es = Cohen’s D for dependent t-tests [<.1 = no effect; .2-.4 = small effect; .5-.7 = intermediate effect; >.8 = large effect]

A number of GPeds reported that a portion of their practice is performed in emergency department (ED) (n = 7 of 51; 13.7%), labor and delivery (L&D) (n = 4 of 51; 7.8%), and newborn nursery (n = 14 of 51; 27.5%) settings. While these GPeds spent less than 40% of their time in these acute care settings (38%, 20%, and 28% respectively), they were more likely to report performing common procedures such as reductions of dislocations (ED), incision and drainage of abscesses (ED), and bag mask ventilation (L&D, newborn nursery). The most common non-ACGME procedure performed was circumcision (newborn nursery). These same GPeds said that they learned these specific procedures either on-the-job by observing colleagues or through formal life support skill classes (Pediatric Advanced Life Support or Neonatal Resuscitation Program).

### How does GPeds procedures education compare to Sawyer’s pedagogical framework?

We present our analyses of how GPeds’ learned procedures during residency through the lens of Sawyer’s model. Table 5 displays relevant themes and quotations for each component of this model.

**Table 5.** GPeds did *not* learn procedures through methods consistent with a mastery learning model: The number (N) and percentage (%) of 51 GPeds who reported that they did not experience components of mastery learning when learning procedures. Representative quotes provide explanation for their lack of experience

Sawyer’s Model Component	Description of Component	Theme	N (%)	Quotations
**Learn (Cognitive Phase)**	Learn about the procedure via readings, videos, and online training. Cognitive tests may be included	***GPeds Did Not Learn or Teach Back Procedures Prior to ‘Doing’***	25 (49.0)	‘*I don’t ever recall being given dedicated lectures to procedures.’* *‘I don’t recall having any textbooks or online resources. I think we did a verbal review with the attending or fellowbefore performing the procedure.’*
**See (Cognitive Phase)**	Demonstration of the skill to a learner with both nonverbal and verbal instruction. Learners are asked to ‘teach back’ to solidify concepts.	***GPeds Did Not Learn or Teach Back Procedures Prior to ‘Doing’***	25 (49.0)	‘*Because of the kind of randomness of when it was your turn to do a procedure, it was often in the moment. It’s like, here it is, jump into the pool and so I didn’t know many resources for education, especially like the Just-in-Time kind of education.’* *‘I was never asked to describe how to do a procedure before doing it on a real person.’*
**Practice (Psychomotor Phase)**	*Formative assessment, which usually entails in-vitro or simulated settings. This phase incorporates deliberate practice.*	***GPeds Did Not Engage in Deliberate Practice***	18 (35.2)	*‘Residency was a big contrast to my nursing training where you would have lectures, labs, and practice before doing a procedure. In nursing, there were multiple dummy trials before you can do it in real life. But as a resident, attendings would say, “You just need to do this”. And if you said “I don’t even know how,” Tthey would say: “Well look it up and do it.”’* *‘No, we did not have any practice or simulation labs. Sometimes at night in the ER we would get out splinting material and splint each other just for practice but it was nothing formal and was not supervised.’*
**Prove (Psychomotor Phase)**	Summative assessment that demonstrate competency and eventually mastery learning. These assessments are typically done in in-vitro or simulated settings.	***GPeds Did Not Prove Competence in Training***	22 (43.1)	***‘****Sure, we were expected to keep a procedure log but no one ever did. So, at the very end, people just fabricated up scenarios.’* *‘You had to log your procedures and you had a minimum quota of logging. But it didn’t even say they had to be observed. You literally could make them up if you wanted to, but I’m obviously not comfortable doing that. But there was definitely not somebody who watched you do it or signed off on you.’*
**Do (Psychomotor Phase)**	Performance of skill on a human being with direct observation, performance-based assessment and feedback.	***GPeds Did Not Participate in Graduated Supervision***	51 (100)	*‘Well, mostly you saw somebody do one, and then you did one under supervision. Sometimes you didn’t even see somebody do one first. It was just like, an attending is telling you and making marks of where it goes and [then doing] the procedure.’* *‘It really was like watching a couple and then doing a couple with the senior resident.’*
**Maintain**	Skill is maintained not only through clinical practice but also supplemented through simulation, procedures logs, individual quality improvement and/or maintenance of certification.	***GPeds Do Not Desire Formal Skill Maintenance***	29 (56.9)	*“Because the scope of practice of different pediatricians is so different, the requirements which you need to be good at are different and you may not be in a setting where you really need to be great at IV placement or laceration repairs. So, I don’t think there is a real need to continue to maintain those skills because medicine is getting pretty compartmentalized*. *‘My initial thought is no, [procedural maintenance] should not be required for [MOC] as we already have enough stuff to keep track of, and it’s too burdensome.’*

#### GPeds did not prove competence in training

None of the participants graduated from residency programs that provided structured summative assessment opportunities for their residents to prove procedural competence. Almost all programs used procedure logs as an indirect measure of competence, sometimes with a minimum number of required encounters per procedure. Some programs used self-reported procedure logs, while others required a supervisor to sign-off that they observed the procedure. Most participants did not feel that procedure logs were defensible evidence of procedural competence, particularly since data could be fabricated.

#### GPeds did not learn or teach back procedures prior to ‘doing’

Very few participants learned about a procedure through lectures or readings prior to being shown the procedure. Some participated in simulations; however, these were most often part of certification programs outside of residency like Pediatric Advance Life Support (PALS) or Neonatal Resuscitation Programs (NRP).

None of the participants reported ‘teaching back’ as part of learning procedures, a fundamental part of the ‘see’ step in Sawyer’s education model. Instead, they reported simply jumping to the ‘do’ portion of the model to perform the procedure on a child with or without supervision.

#### GPeds did not engage in deliberate practice

With the exception of PALS or NRP courses taken during or after training, the participants did not commonly engage in deliberate practice during training, whether it was in a simulated environment or during training in the clinical environment. Some reported receiving feedback and coaching while performing procedures on live patients, but were not required to prove that they were competent prior to performing the procedure in the clinical setting. Others simply avoided performing procedures they did not feel competent to perform.

#### GPeds did not participate in graduated supervision

Participants did not mention graduated supervision as part of their learning. Some participants took almost complete responsibility for learning procedures on their own. Others mentioned that, depending on their future professional plans, they pursued additional opportunities through which to learn and practice procedures outside of the residency program such as moon-lighting, elective rotations, or external courses. Even in those settings, they reported not learning these skills through graduated supervision.

#### GPeds did not maintain all skills and do not desire formal skill maintenance

Only two participants pursued post-graduate training workshops in procedural skills. A few GPeds commented that they have let their life support certifications lapse since these were not needed in their current medical practices.

When asked how they would ‘relearn’ a procedure if they happened to relocate or change job types, most said that they would prefer to shadow or be observed by peers as opposed to attending training workshops. They wanted their employer or local medical center to ensure they were credentialed and/or to complete ‘check-offs’ for competency. Furthermore, most participants voiced concerns about introducing formal procedural competence assessments as a component of maintenance of certification (MOC). Participants stated that since GPeds’ scope of practice varied so broadly, governing medical bodies and boards could not fairly implement requirements. In addition, they reported that requiring procedural MOC would be costly, burdensome, and add to the ever-growing list of administrative requirements that occupy a physician’s time. Above all, GPeds believed that procedural MOC is unnecessary since not all procedures are relevant to the practice of all GPeds.

## Discussion

Using Sawyer’s framework as a lens, our results suggest that although GPeds had some experience with most required procedures, they never had the opportunity to learn *all* these procedures to the level of competence during training. Moreover, residency training programs did not formally assess procedural competence, so many GPeds graduated without documented evidence or the self-perception that they were proficient at performing the procedures asked them about in this study. Recent studies are consistent with these findings. For example, a recent survey of graduating pediatric residents showed that 33.3% may not have completed one or more of the required procedures successfully in training[31]. Program directors also have reported pediatric residents fail to achieve competence in procedures such as venipunctures, neonatal endotracheal intubations, and administering immunizations[32]. Furthermore, although participants reported completing procedure logs, such documentation is designed for program evaluation and not for providing evidence of individual competence [24,33,34].

From prior studies, we know GPeds believe procedures are an important part of their residency education for five reasons. They want to be: 1) adaptable to potential changes in their type of practice or practice location, 2) prepared for emergencies in which a life-saving procedure is needed, 3) sufficiently knowledgeable to describe procedures to patient families, 4) able to perform the procedure in a situation where they were too far from a PTC, and 5) align with conceptualizations of GPeds in the formation of their professional identity[24]. We also know that not learning procedures during residency training impedes GPeds from performing procedures in practice[24]. Our data showed this to be particularly salient for certain high-risk, low frequency procedures (endotracheal intubation, lumbar puncture, umbilical catheter placement) that GPeds reported feeling less prepared to perform. Not learning and performing procedures, therefore, made maintenance of skills nearly impossible. In fact, most GPeds suggested that they had not maintained such skills and would have to relearn them if required to demonstrate competence during formal MOC assessment or in practice. Together, these results may at least partially explain GPeds disinclination to perform some ACGME procedures in practice.

Our results also call into question the need for GPeds to learn the specific 13 procedures currently required by the ACGME. Pediatric healthcare is evolving. Trends toward the use of procedural specialists or procedure technicians, referrals of procedures to pediatric sub-specialists or to emergency departments and urgent cares, and not receiving adequate procedures education during pediatric residency are all existing barriers to GPeds performing these procedures in their current practice [35,36]. With this in mind, perhaps GPeds do not need to become proficient in all of these procedures during residency training. For example, in this and in other studies, it has been reported that in private practice or office-based settings, the administration of immunizations is done primarily by medical assistants and nurses rather than GPeds[23]. All GPeds interviewed for this study said that they had referred at least one procedure to an emergency department or subspecialist. This suggests that many of these procedures have likely fallen out of the GPed’s scope of practice and that graduate medical education should adapt accordingly.

### How should pediatric residency programs teach procedures?

We used Sawyer’s framework as a gold-standard lens to evaluate procedures education because of its comprehensive focus on procedural steps and skill maintenance to prevent decay. Through this lens and from our GPeds responses, implementing mastery-based learning more broadly into pediatric procedures education would not necessarily be beneficial given the investment of time required for mastery learning and the fact that some procedures seem to have fallen out of GPeds’ scope of practice. We also found that the setting in which the GPeds in our sample practiced, the type of patients they saw in that environment, and the other types of providers that were available all determined the specific procedures they needed to perform. Study participants reported being significantly less prepared currently as compared to graduation from residency on 5 of 13 procedures (neonatal endotracheal intubation, umbilical catheter placement, lumbar puncture, simple laceration repair, and peripheral intravenous catheter placement), simply because the patients they see in their current practice settings do not need these procedures. Other procedures from our list (giving immunizations or venipuncture) may have been performed in their current practice settings, but were often delegated to other healthcare practitioners. Finally, there seem to be a few procedures, such as splinting, that GPeds were reluctant to perform due to the cost of maintaining supplies in an office-based setting, and others that they do perform that are currently not taught in pediatric residencies.

A critical question as we consider training modifications becomes: should procedures education in pediatric residencies consider the type of future practice in which the general pediatrician plans to work? Track-based education or customized procedural training may help to meet the needs of future general practitioners, without overwhelming an already dense pediatric program curriculum. Such tailored education could differentiate procedures education for individuals who plan on various subspecialty fellowships or practice types. For example, those going into critical care or who plan to practice in a rural, military, or global setting would need to master emergent care procedures such as airway management and rapid intravenous access; whereas those planning on primary care practices in urban or suburban settings would need to master office-based procedures such as bladder catherization [20,37]. Within each track, mastery of specific required procedures, through clinical practice and/or simulation, and surface-level coverage–learning without the expectation of eventual competent performance–of other less relevant skills would streamline procedures education. This could then ensure that the practitioners are learning procedures relevant to their future practice and that those specific procedures are mastered in order to promote maintenance over time and reduce the associated medical errors or adverse patient outcomes that comes from deskilling [38,39]. While low clinical volumes may impact the ability to maintain skills, alternative methods of learning, such as rolling refreshers (e.g., just-in-time in situ training sessions), procedural simulations, and semi-frequent post-graduation skill assessment in practice-specific procedures could be adopted [40–42].

### Further implications of adapting procedural education requirements

If the ACMGE and residency programs modify procedure requirements to the future needs of pediatric residents or if the ACGME eliminates procedural requirements altogether, adaptations will be needed for core educational requirements. Further research using practice pattern analyses could be used to determine which procedures are relevant to each type of practice and each pediatric subspecialty. This would also entail investigating the depth to which each required procedures need to be learned for various types of clinical settings. In addition, changes to procedural requirements would have significant implications on formalizing standards of practice for how these procedures are referred and to whom, highlighting the role that GPeds play in the medical homes for their patients[43].

The primary limitation in this study was that participants’ responses depended on recall of their training experiences. Most (94%) of our interviewees’ have been in practice for thirty years or less and had some experience during training with all of the procedures we inquired about. Some of those who had been out of training for a long time had some difficulty in answering questions about when and where they learned specific procedures. However, almost none had difficulty in recalling ‘how’ or ‘how well’ they learned each procedure. In addition, the majority of the sample GPeds worked in the central region of the country and completed residency programs in that region (many in the State of Ohio). Accordingly, our findings may not be completely generalizable to other regions of the U.S. or other countries. Finally, GPeds were not asked their beliefs about the optimal methods of procedural learning, and thus, their suggestions for how best to deliver procedures education were not offered.

## Conclusions

Accreditation bodies such as the ACGME establish rules and regulations designed to guide residency programs. For pediatrics programs, they have historically recommended or required that residents demonstrate competence in clinical procedures prior to graduation. The GPeds interviewed in our study said that they never learned many of the currently required procedures, nor were they required to demonstrate competence through formal assessment of their skills. This lack of training may partially explain why GPeds infrequently perform these procedures and why they are more likely to refer them to specialists. An alternative, and equally compelling explanation, is that these required procedures simply may not be needed in the practice of most GPeds. Further research is needed to establish the procedural scope of practice for GPeds and pediatric subspecialists. Once this is achieved, procedures education can be tailored to the needs of the pediatrician based on their future practice plans.

## How General Pediatricians Learn Procedures: Implications for Training and Practice

**Appendix A. Interview Guide and Closed Ended Questions**

*The Accreditation Council for Graduate Medical Education (ACGME) requires thirteen procedures in which residents must demonstrate competence prior to graduation. These procedures are listed in the Table below.*

**Table. The 13 procedures recommended by the 2013 ACGME**

**Table:**

Bag-Valve Mask Ventilation Neonatal Endotracheal Intubation Umbilical Catheter Placement	Lumbar Puncture Simple Laceration Repair Incision & Drainage of Abscess Reduction of a Dislocation Temporary Splinting of a Fracture	Giving Immunizations Bladder Catheterization Peripheral Intravenous Catheter Placement Venipuncture Removal of a Foreign Body

### Procedural Training Experience

1. Thinking back to your residency training, describe the education you received on each of the 13 ACGME procedures?

A.Where did your learning of these procedures take place?

B.Who taught you these procedures?

C.How did you learn how to perform these procedures?

i. Were these lectures (cognitive) or psychomotor based (hands-on) experiences?

ii. Were you formally tested on being competent in these procedures?

2. How prepared did you feel to perform these procedures upon graduation?

*UnpreparedSomewhat preparedWell prepared*

**Current Procedural Practice and Skill Maintenance**

Let us discuss your typical daily practice as a general pediatrician.

3. Tell me about your current practice setting?

4. What procedures do you personally perform in your practice?

5. Describe any post-graduate procedural training courses, workshops, skill sessions in which you participated? Why did you take these?

6. Some specialties, like surgery require procedure logs to qualify for sitting for the initial board certification exam and for maintenance of certification. In addition, most hospitals and health plans require documentation of maintenance of PALS/CPR skills over time in practice. However, some of the pediatric procedures in the list provided do not require such documentation.

A. Do you think that documentation should be required? Why or why not?

B. Do you think this documentation of competency for these procedures is important?

7. How prepared you are to perform these procedures today?

*UnpreparedSomewhat preparedWell prepared*

**DEMOGRAPHIC QUESTIONS**

1)Did you complete residency training in pediatrics or medicine-pediatrics?

1. Yes
2. No

2)In what year did you complete your residency training?

3)At what institution did you complete your residency training?

4)In what type of setting do you practice? Please report the % of time spent in the each setting.

- Office/private practice
- Federally Qualified Health Center
- Newborn Nursery
- Urgent Care
- Emergency Department
- Hospital/Medical Center
- Labor and Delivery
- Other

5)Are there other providers in your practice (MD, NP, PA, etc)

1. Yes

B.No

6)Do you work full time?

1. Yes
2. No

7)Did you complete subspecialty/fellowship training?

1. Yes
2. No

8)If yes to #7, what was your subspecialty or fellowship training in?

9)What is your gender?

A.Female

B.Male

10)How many years have you been practicing as a general pediatrician?

1. <5
2. 5–10
3. 11–20
4. 21–30
5. >30

11)Where do you refer procedures?

- Emergency Department
- Urgent Care
- Hospital-based specialist
- Outpatient based specialist
- I do not refer out any procedures

12)How far from your practice is the nearest pediatric medical center (in miles)?
